# Post-Traumatic Distress and Burnout Among Chinese School Teachers: The Mediating Role of Forgiveness

**DOI:** 10.3389/fpsyg.2021.642926

**Published:** 2021-07-30

**Authors:** Yabing Wang, Man Cheung Chung, Siqi Fang

**Affiliations:** ^1^School of English Education, Guangdong University of Foreign Studies, Guangzhou, China; ^2^Faculty of Education, The Chinese University of Hong Kong, Hong Kong, China; ^3^Department of Social and Behavioral Sciences, City University of Hong Kong, Hong Kong, China

**Keywords:** PTSD, psychiatric co-morbidity, burnout, forgiveness, work-related stressors

## Abstract

**Purpose:**

Teachers’ mental health is concerning due to high stress at work. Its association with job-related stressors has been well-documented. Little is known; however, about how traumatic life events and trauma reactions might contribute to their psychological distress. This paper is to explore whether Post-traumatic Stress Disorder (PTSD) following past traumatic event would predict burnout and psychiatric co-morbidity among Chinese k-12 school teachers and whether this prediction would be mediated by forgiveness after controlling for work-related factors.

**Methods:**

Two hundred and seventy-nine Chinese teachers (*F* = 223, *M* = 56) from primary and secondary schools completed demographic information, Post-traumatic Stress Disorder Checklist for DSM-5 (PCL-5), Heartland Forgiveness Scale (HFS), General Health Questionnaire-28 (GHQ-28), Maslach Burnout Inventory-Educator’s Survey (MBI-ES), and a series of measures assessing work-related factors.

**Results:**

Structured equation modeling (SEM) showed that after controlling for work-related factors, PTSD following past trauma was positively associated with burnout and general psychological problems but negatively associated with levels of forgiveness. Forgiveness carried the impact of PTSD onto burnout rather than general psychological distress.

**Conclusion:**

To conclude, regardless of the level of stress experienced from working in school, primary and secondary teachers with PTSD from past trauma found it more difficult forgiving which in turn could affect their levels of burnout.

## Introduction

Teaching is a highly stressful profession (Skaalvik and Skaalvik, 2017) and can lead to a wide range of physical and mental health problems among teachers (Kyriacou, 1987, 2001; Grayson and Alvarez, 2008). One commonly reported outcome is burnout, composed of tripartite symptom clusters, namely, emotional exhaustion, depersonalization, and a reduced sense of personal accomplishment (Maslach et al., 1996). Individuals with burnout often suffer from physical illnesses, sleep disturbance, depression, anxiety, substance abuse (Bakker et al., 2014) and experience work or family conflict (Swider and Zimmerman, 2010). In addition to these personal ramifications, substantial costs can be incurred to schools due to turnover rate (Liu and Onwuegbuzie, 2012), absenteeism (Van Dick and Wagner, 2001), and reduced job satisfaction and performance (Maslach et al., 2001; Skaalvik and Skaalvik, 2009).

Though burnout could manifest in many occupations, it is particularly prevalent and detrimental in educational settings due to the day-to-day work stressors teachers experience (Iancu et al., 2018). Thus, numerous studies have investigated the consequences of it for both teachers and students. The increase of it could diminish not only teachers’ thoroughness of classroom preparation and health (Billingsley and Bettini, 2019), but also students’ perceived teacher autonomy support and instructional quality, intrinsic motivation, actual engagement in classroom activities, and academic achievement (Shen et al., 2015; Madigan and Kim, 2021).

In terms of risk factors, whilst individual factors (e.g., personal characteristics and emotional intelligence) were found to contribute to burnout among teachers (Chan, 2013), little is known whether PTSD (i.e., a psychiatric disorder triggered by traumatic events manifested by a range of symptoms, such as avoidance of trauma reminders and intrusion of trauma images) would contribute to it, along with other psychological symptoms, and what possible factors might mediate the influence of PTSD on distress outcomes.

Though the Job Demands-Resources (JD-R) model (Demerouti et al., 2001) viewed job demands as the primary source of burnout, an increasing number of studies tried to explain work experience from the perspective of personal demands and/or resources grounded on theoretical models, such as the conservation of resources (COR) model (Hobfoll, 2002) and person-environment fit model (Caplan, 1975). These models provide common insights such that personal strain could interfere with work experience and adaptation. Thus, it is reasonable to speculate that PTSD, as psychological distress and strain, could erode and compromise personal resources and job performance.

The link between PTSD, burnout and psychiatric co-morbidity among teachers is plausible, given that the majority (approximately 80%) of the community will experience at least one traumatic event during the entire life (Breslau, 2009), of whom about 6–12.3% would develop PTSD (Breslau, 2009) alongside depression and anxiety (Chung and Reed, 2017). PTSD was linked with burnout among service professions, such as firefighters (Katsavouni et al., 2016) and nurses (Mealer et al., 2009). However, only one study has investigated this link among teachers and found that the impact of violence on burnout was mediated by PTSD symptoms (Rojas-Flores et al., 2015).

Past trauma can affect constituents of oneself, change one’s internal world and identity (Berntsen and Rubin, 2006), challenge interpersonal functioning, and meaningful relationships (Briere and Runtz, 2002). Following the logic, one would speculate that forgiveness engagement and manifestation would be difficult tasks due to the entrapment of trauma and grudge against the transgressors (Werdel and Wicks, 2012). Additionally, specific manifestations of PTSD, such as blame on others, angry feelings as well as constant negative mood and cognition (e.g., no one is trustworthy), could also lead to difficulty in forgiving (Noll, 2005).

As a result, according to the Stress-and-Coping framework (Worthington and Scherer, 2004), unforgiveness, in this case ensuing PTSD as a stress-reaction and attempt to cope, can engender behavioral and psychological mal-adaptations (Bono et al., 2008). It has been related to worse mental health (Berry and Worthington, 2001) and increased burnout among teachers (Chan, 2013). These studies imply that forgiveness could act as a mediator between PTSD and distress outcomes. In fact, the mediating role of forgiveness has been demonstrated in several studies (Snyder and Heinze, 2005; Solomon et al., 2009). Notwithstanding that, whether it would mediate the impact of PTSD from past trauma onto burnout and psychiatric co-morbidity among teachers is unclear.

**FIGURE 1:**
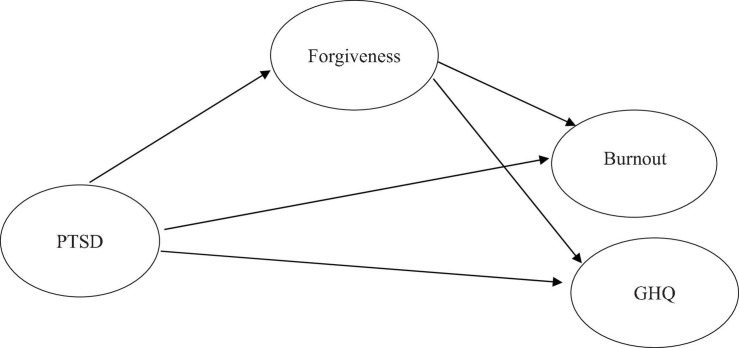
Hypothesized model with forgiveness mediating PTSD from past trauma, burnout, and mental health.

This study aimed to explore whether (1) PTSD would predict burnout and psychiatric co-morbidity; (2) forgiveness would be associated with PTSD, burnout and psychiatric co-morbidity; (3) forgiveness would mediate the relationship between PTSD and distress outcomes among Chinese school teachers (see Figure 1). This investigation, however, needed to take account of job-related factors e.g., students’ behavioral problems, heavy workload, role conflict and ambiguity, and perceptions of the school climate since they have proved to impact teachers’ stress (see a review: Maslach, 2003).

After controlling for work-related factors, we hypothesized that (see Figure 1):

(1) PTSD symptom severity would be positively correlated with levels of burnout and psychiatric co-morbidity.
(2) Forgiveness would be negatively associated with PTSD, burnout, and psychiatric co-morbidity.
(3) Forgiveness would mediate the path between PTSD and distress outcomes.

## Materials and Methods

### Procedure and Participants

A cross-sectional non-experimental survey was conducted. Anonymous self-report questionnaires were administered in order to obtain data. Ethics approval for the current study was obtained from Survey and Behavioral Research Ethics Committee of the affiliated university (approval number: EDU2017-112). With convenience sampling, teachers from four Chinese public schools were approached in a meeting after permission from school principals. After debrief of the research, those willing to participate were asked to return the completed questionnaire within 1 week. 279 (*F* = 223, *M* = 56) full-time primary (*n* = 68) and secondary school teachers (*n* = 211) participated. Informed consent was signed by every participant. The purpose of the study was presented and they were informed that their responses would be anonymous and used only for research purposes. They were entitled to withdraw from the study without any penalty even after signing the consent form. The inclusion criteria were: (1) full-time teachers, (2) age 18 or above, and (3) ethnically Chinese. 780 questionnaires were distributed in total (response rate 36%). All the questionnaires were back translated.

### Measures

A demographic page was administered to collect information on participants’ gender, age, years of teaching, marital status, education background, and whether they were primary, secondary or form teachers. A form teacher is someone with assigned responsibilities e.g., overseeing the academic progress of students in a specific class, and liaising with parents. These responsibilities are additional to classroom teaching as well as moral education.

Post-traumatic Stress Disorder Checklist for DSM-5 (PCL-5) (Weathers et al., 2013) aims to assess PTSD. The first part covers a range of past traumatic life events that participants may have experienced. If more than one has been experienced, the most traumatic one is identified. Based on the DSM-5 criteria, the second part assesses PTSD symptoms related to the most distressing traumatic event (e.g., “In the past month, how much were you bothered by repeated, disturbing, and unwanted memories of the stressful experience?” “…by loss of interest in activities that you used to enjoy?”). The symptoms are grouped into four clusters: intrusion, avoidance, negative mood and cognition, as well as alterations in reactivity and arousal. PTSD symptoms are measured using a four-point Likert scale: 0 = not at all, 1 = a little bit, 2 = moderately; 3 = quite a bit; 4 = extremely. This scale has demonstrated excellent psychometric properties (Blevins et al., 2015). Based on the sample of this study, the Cronbach’s α for PTSD total was 0.95.

Heartland Forgiveness Scale (HFS) (Thompson et al., 2005) contains three subscales: forgiveness of self, others, and situations (e.g., “I continue to punish a person who has done something that I think is wrong,” “If I am disappointed by uncontrollable circumstances in my life, I continue to think negatively about them”) on a 7-point scale (1 = almost always false of me to 7 = almost always true of me). A high total score represents a high level of forgiveness. Good internal consistency ranging from 0.84 to 0.87 and test-retest reliability (3-week, *r* = 0.83; 9 month *r* = 0.77) have been reported (Yamhure-Thompson and Snyder, 2003). In this study, the Cronbach’s α was 0.80.

The educators’ version of Maslach Burnout Inventory (MBI) (Maslach et al., 1996) was used to measure teachers’ burnout (e.g., “I feel emotionally drained from my work,” “I’ve become more callous toward people since I took the job”) using a 7-point rating (0 = never to 6 = every day). It has been validated at different educational levels (Kokkinos, 2007). The MBI is composed of three subscales: emotional exhaustion, depersonalization, and reduced sense of personal accomplishment. Reverse-coding is applied to the rating of personal accomplishment. Based on this sample, the Cronbach’s α for the total score was 0.90.

The General Health Questionnaire-28 (GHQ-28) aimed to measure teachers’ general psychological problems. In PTSD research, GHQ-28 has been recommended to estimate general co-morbid symptoms (Chung and Chen, 2017). The scale comprised four subscales: somatization, anxiety, social dysfunction, and depression. In this study, the Cronbach’s α for the total score was 0.94.

Work-related stressors were measured using a series of questionnaires. Workload and student misbehavior were measured using Teacher Stress Inventory (Boyle et al., 1995) on a 5-point scale (0 = no stress to 4 = extreme stress). Cronbach’s α were 0.74 for workload and 0.85 for student misbehavior. Role conflict and ambiguity were measured using Role Conflict and Role Ambiguity Scale (Rizzo et al., 1970) on a 5-point scale (1 = strongly disagree to 5 = strongly agree). Cronbach’s α were 0.79 for role conflict and 0.86 for role ambiguity in the current study. Perception of school climate was measured using Revised School-Level Environment Questionnaire (Johnson et al., 2007) on a 5-point rating scale (1 = strongly disagree to 5 = strongly agree). It yields five factors: collaboration, student relations, school resources, decision making and innovation, and was validated on primary and secondary levels. Based on this sample, the Cronbach’s α for the total score was 0.85.

### Data Analysis

Descriptive analyses were used to describe demographic data. Correlation coefficients were used to show the relationship between teachers’ demographics, burnout and psychiatric co-morbidity. Multivariate analyses were used to compare mean differences across diagnostic groups, and structured equation modeling (SEM) used to examine the relationship among the latent variables of PTSD, forgiveness, and distress outcomes. We used the maximum likelihood (ML) estimation in Mplus 7.0 to test the hypothesized model with indices as follows: Comparative fit index (CFI) and Tucker-Lewis index (TLI) with 0.90 and 0.95 or above suggesting good and excellent fit, respectively; Root mean square error of approximation (RMSEA) and standard root mean-square residual (SRMR) with 0.06–0.08 and 0.06 or below suggesting an acceptable fit and excellent fit respectively; χ^2^ significance test as well as the ratio of χ^2^/df less than 3 was recommended (MacCallum et al., 1996).

PROCESS was to verify the mediation analysis. Confidence intervals were used to address the problem of bias due to non-normalized sampling distributions of mediating effects. The bootstrapping sampling (*n* = 5,000) distributions of indirect effects were produced by drawing cases from the original sample and generating indirect effects in the resamples (Hayes et al., 2017). Point estimates and confidence interval (CI; 95%) were used for estimating indirect effects. When the confidence interval does not contain zero, indirect effects would be regarded as significant. Missing data was estimated by regression imputation. This method is valid given that the missing response rate was less than 5%.

## Results

### Demographic Information

The mean age of the 279 teachers was 35 (*SD* = 7.25) with 12.39 (*SD* = 8.63) as the mean years in teaching; 38% were form teachers. Most of the sample were married (80.6%) and held university degree (91%).

### Differences Between Diagnostic Groups

Focusing on PTSD from past trauma, 90% of participants reported that they had experienced trauma, including sudden death of close ones (74%), life-threatening illness (24%) and school violence (23%). The average number of past trauma was 2.89 (*SD* = 3.42). On average, the most distressing trauma happened 53 months ago (*SD* = 48.49). Using the PTSD diagnostic standards (full PTSD: 1 item about avoidance and intrusion AND 2 items about negative mood and cognition and hyperarousal scoring no lower than 2; partial-PTSD: 1 item about one of the four subscales scoring no lower than 2), 12% met the threshold of full-PTSD (exhibiting four symptom clusters); 39% partial-PTSD (exhibiting some symptom clusters), and the rest (49%) no PTSD.

There were significant differences between diagnostic groups in all psychological constructs except role ambiguity and workload (see Table 1 for group comparison). Compared to the no-PTSD group, teachers with full-PTSD reported higher levels of burnout, psychiatric co-morbidity, role conflict, student misbehavior and lower levels of forgiveness as well as perceptions of school climate. Compared to those with partial-PTSD, the full-PTSD group reported significantly higher on burnout, psychiatric co-morbidity, role conflict, and student misbehavior.

**TABLE 1:** The means and standard deviations of forgiveness, burnout, psychiatric co-morbidity, and work-related stressors for the full-PTSD, partial-PTSD, and no-PTSD groups.

	Full-PTSD *N* = 33		Partial-PTSD *N* = 110		No-PTSD *N* = 136		*F*(2,276)
	Mean	SD	Mean	SD	Mean	SD	
Forgiveness	80.6	7.4	84.9	14.4	91.2	12.0	12.9***
Burnout	55.2	16.0	43.6	15.2	37.5	14.6	19.3***
Co-morbidity	64.4	51.2	51.0	9.6	46.9	10.1	37.2***
Role conflict	36.1	7.4	33.0	7.4	31.3	7.8	5.7**
Role ambiguity	20.0	6.8	17.8	7.0	17.8	6.4	1.6
Workload	5.7	1.7	4.8	1.9	4.6	1.9	4.5
Student misbehavior	8.2	5.0	6.3	3.9	5.7	3.8	5.1**
School climate	71.7	9.0	74.2	8.8	76.9	9.8	5.2**

Co-morbidity: psychiatric co-morbidity; ***p < 0.001; **p < 0.01.

### Correlation and Mediation Analyses

Correlation analyses were used to examine whether demographic information was associated with distress outcomes. Those significant background variables would be controlled for in the subsequent SEM analysis. Given that trauma type has been shown to influence distress, traumatic events were classified into interpersonal and non-interpersonal types. To adopt the definitions used in literature (Westphal et al., 2013; Alisic et al., 2014), interpersonal trauma includes assault (either physical or sexual) from another person with or without a weapon, and interpersonal violence (e.g., domestic violence, intimate partner violence, school violence) whereas non-interpersonal trauma results from non-intentional causes (e.g., car accident, life-threatening disease).

After adjustment with Bonferroni with the *p* = 0.002, none of the demographic variables (including trauma type) was associated with burnout or psychiatric co-morbidity. However, all work-related stressors were correlated with the two outcomes (| *r|* s ranging from 0.19 to 0.41; see Table 2 for correlation details).

**TABLE 2:** Correlation matrix among subscales of PTSD, forgiveness, burnout, and psychiatric co-morbidity.

	1	2	3	4	5	6	7	8	9	10	11	12	13	14
1. Intru	1													
2. Avoid	0.53**	1												
3. Nega_m	0.74**	0.53**	1											
4. Hyper	0.73**	0.49**	0.82**	1										
5. Forself	−0.21**	–0.09	−0.18**	−0.20**	1									
6. Forthers	−0.24**	−0.16**	−0.26**	−0.28**	0.29**	1								
7. Forsit	−0.26**	−0.21**	−0.30**	−0.31**	0.44**	0.55**	1							
8. EE	0.38**	0.24**	0.42**	0.40**	−0.23**	−0.32**	−0.35**	1						
9. DP	0.36**	0.17**	0.46**	0.40**	−0.23**	−0.40**	−0.36**	0.68**	1					
10. RPA	0.19**	0.10	0.21**	0.26**	−0.17**	−0.36**	−0.39**	0.33**	0.38**	1				
11. Soma	0.35**	0.18**	0.28**	0.34**	−0.20**	−0.18**	−0.19**	0.43**	0.25**	0.26**	1			
12. Anxi	0.46**	0.24**	0.43**	0.47**	−0.27**	−0.29**	−0.29**	0.48**	0.37**	0.28**	0.70**	1		
13. So_dys	0.38**	0.25**	0.39**	0.38**	−0.18**	−0.15*	−0.16**	0.42**	0.32**	0.27**	0.51**	0.63**	1	
14. Depres	0.41**	0.30**	0.46**	0.43**	−0.18**	−0.22**	−0.25**	0.45**	0.40**	0.25**	0.46**	0.58**	0.62**	1

Intru, Intrusion; Avoid, Avoidance; Nega_m, Negative mood and cognition; Hyper, Hyperarousal; Forself, Forgiveness of the self; Forthers, Forgiveness of others; Forsit, Forgiveness of situation; EE, Emotional exhaustion; DP, Depersonalization; PA, Reduced personal accomplishment; Soma, Somatic; Anxi, Anxiety; So_dys, Social dysfunction; Depres, Depression. **p < 0.01.

**FIGURE 2:**
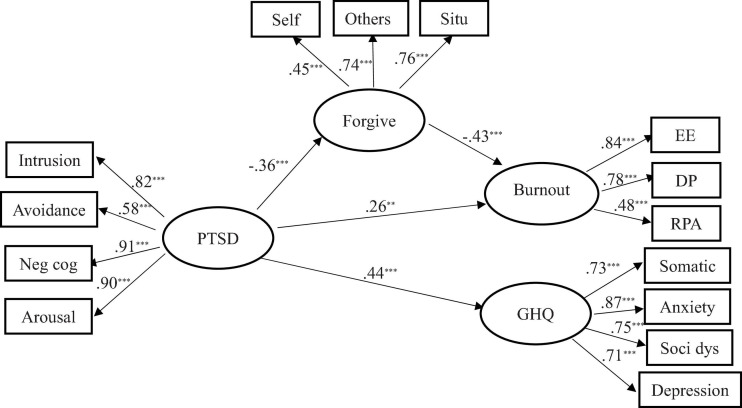
Significant standardized results of regression weights and factor loadings.

Structured equation modeling (SEM) was used to examine the hypothesized model with work-related factors treated as covariates. The correlation matrix among all the indicators of PTSD, forgiveness, burnout, and psychiatric co-morbidity can be found in Table 2. All indicators of latent constructs significantly loaded onto them mostly at *p* < 0.001. Estimation of this model generated acceptable fit: χ^2^ = 250; *df* = 121; CFI = 0.94; TLI = 0.91; RMSEA = 0.06; SRMR = 0.04. PTSD was significantly correlated with forgiveness and distress outcomes with path coefficients ranging from 0.26 to 0.44. Forgiveness was correlated with burnout (effect size = 0.43) but not psychiatric comorbidity (see Figure 2). PROCESS examined direct and indirect effects by bootstrap mediation analysis. Results showed that forgiveness partially mediated the relationship between PTSD and burnout (β = 0.07; Boot LLCI = 0.03; Boot ULCI = 0.11) (see Table 3).

**TABLE 3:** PROCESS results of mediation effect of PTSD on burnout with work-related factors as covariates.

	β	SE	LLCI	ULCI	BootSE	BootLLCI	BootULCI
**Outcome (burnout)**							
**Direct effect**							
PTSD- burnout	0.20	0.06	0.08	0.32	–	–	–
PTSD- forgiveness	–0.25	0.06	–0.36	–0.13	–	–	–
Forgiveness burnout	–0.27	0.06	–0.38	–0.15	–	–	–
**Indirect effect *via* forgiveness**							
	0.07	–	–	–	0.02	0.03	0.11

*SE, bootstrapped standard errors; 95% CI bias corrected-confidence interval.*

## Discussion

This study explored whether PTSD following past trauma would be related to burnout and psychiatric co-morbidity among Chinese school teachers and whether this link would be mediated by forgiveness over work-related factors. All hypotheses were partially supported. After controlling for these factors (workload, student misbehavior, role conflict and ambiguity, and perceptions of school climate), PTSD symptom severity was positively associated with levels of burnout and psychiatric co-morbidity; forgiveness was negatively correlated with PTSD and burnout rather than psychiatric co-morbidity. Teachers who scored high on PTSD were less forgiving which was associated with burnout only.

The prevalence rate of PTSD based on this sample is one of the first pieces of evidence indicating the detrimental effect that past traumatic events can have on burnout and psychological distress among these teachers. The 12% for full-PTSD is compatible with the top end of the prevalence rate reported in literature on general population (Breslau, 2009) but lower than that (19.9%) among teachers exposed to community violence (Rojas-Flores et al., 2015). These differences could result from the measures used. E.g., while the Los Angeles Symptom Checklist was used to assess PTSD based on DSM-IV criteria in the preceding study (Rojas-Flores et al., 2015), PTSD Checklist for DSM-5 was used in this study. Also, cultural differences could have contributed to differences in the prevalence rate. The teachers exposed to community violence were selected from El Salvador, whilst ours were from China. PTSD symptoms, especially avoidance, can be expressed differently across cultures, although there is evidence suggesting that intrusive symptoms may be universal (Macdonald et al., 2013).

This full-PTSD diagnosis should not overshadow the importance of partial or sub-syndromal PTSD which occurs along a spectrum of normal to abnormal stress reactions. Front-line teachers with a partial-PTSD diagnosis may also face trauma symptoms interfering with physical and psychological functioning; their clinical care cannot be neglected.

Focusing on the relationship between PTSD and burnout, Bakker et al. (2014) job demands-resources model (JD-R model) suggests that a lack of personal resources can contribute to burnout. Trauma could have affected teachers’ personal resources because their self-capacity (Briere and Runtz, 2002) to, e.g., tolerate, control or regulate stress from job demands might have been altered leading to burnout. In short, in agreement with the JD-R model, whilst it is important to focus on the shortage of personal resources as important factors for developing job strain, how these resources or the sense of self have been “altered” by the trauma, as opposed to the lack of them, also needs to be considered.

The positive relationship between PTSD and psychiatric co-morbidity agrees with epidemiological studies suggesting that PTSD is not a disconnected clinical syndrome. Rather, it overlaps and co-exists with other disorders, with anxiety and depression as the most common co-morbid symptoms (Breslau, 2009). This association could mean that teachers with pre-existing psychiatric disorders were at risk of developing PTSD (Ginzburg et al., 2010). It could also mean that the trauma induced multiple psychiatric symptoms.

These results on the negative association between PTSD and forgiveness among teachers seemed to have contradicted post-traumatic growth literature suggesting that traumatized individuals can experience a phenomenological transformation where their forgiving disposition post-trauma is heightened by spiritual transformation (Janoff-Bulman, 2004). Contrastingly, the teachers in this sample have transformed into individuals who had restricted ability to forgive (Noll, 2005). These results might reflect, according to the self-trauma model (Briere, 1996), aspects of distorted cognitions or schematic self-change, others and the world after trauma (Janoff-Bulman, 1992). They might endorse maladaptive coping strategies, e.g., blaming themselves or others (Janoff-Bulman, 1992). Distorted cognitions and maladaptive copings would likely keep forgiveness at bay, although this speculation has yet to be verified.

The results on the negative correlation between forgiveness and burnout imply that difficulty in releasing vengeance and anger could relate to chronic emotional exhaustion, emotional disengagement, and a decline in feelings of accomplishment at work. Further analysis showed that all three subscales of forgiveness were significantly associated with all symptom clusters of burnout with correlation values ranging from −0.17 to −0.40. This suggests that forgiveness is a particularly important issue for teachers because their job requires interpersonal relationships or sensitivity with students and their parents, colleagues and school administrators. The fact that all forgiveness types and burnout clusters were correlated suggest that having a general unforgiving attitude rather than having specific unforgiving types (e.g., unforgiving of oneself or others) influenced burnout. This contradicts the claim that types of forgiveness are related to specific distress outcomes (Witvliet et al., 2004).

Furthermore, mediation results suggest that this general unforgiving attitude influenced PTSD to burnout only rather than general psychological problems. Separate trauma responses have emerged depending on type of distress outcome. Focusing on teachers’ emotional work distress and job accomplishment, both the severity of past trauma and difficulty in forgiving played a role. However, focusing on general psychological distress rather than job-related distress, the severity of past trauma appeared to have a specific impact suggesting specific cognitive or emotional processes exist for explaining different distress reactions. This has been supported in literature (Chung and Reed, 2017). This also echoes the connectionist system hypothesis arguing that a particular situation, such as a specific traumatic event, can induce a particular cognitive-affective unit of reactions, burnout or general psychological distress in this study alongside with distinctive tendencies of behavior responses, i.e., reduced ability to forgive (Mischel and Shoda, 1995).

These mediation results have important implications for psychotherapeutic interventions for teachers who have experienced trauma and/or suffer from burnout. Previous studies have investigated the effectiveness of spirituality intervention (Chirico, 2017b; Chirico et al., 2020) and other positive interventions (e.g., mindfulness; see Iancu et al., 2018 for a meta-analytic review) in alleviating teachers’ stress and concerning burnout (Chirico, 2017a). Built on this study, future prevention and intervention programs should, in part, aim to empower front-line teachers to adopt an attitude of forgiveness, which is potentially more cost-effective since it could be self-practiced. In so doing, they might buffer against burnout. Systematic evidence-based studies are, however, needed to verify this, although forgiveness therapy has been shown to be effective in improving psychological well-being (Reed and Enright, 2006; Chirico and Magnavita, 2020).

Notwithstanding this, the lack of mediational effects on the path between PTSD and psychiatric co-morbidity suggest that addressing issues pertaining to past trauma should constitute part of the therapeutic program alongside the empowerment of forgiveness. In other words, the results advocate the important integration between positive psychology and conventional trauma-focused types of intervention for teachers with trauma.

There are limitations in the study. Firstly, low response rate restricted the representativeness of the results. E.g., teachers with severe PTSD might have avoided, as part of their symptoms, participation in the study. Secondly, the study did not examine whether cultural beliefs might interact with PTSD symptoms to influence distress outcomes. Literature shows that ethnicity of which cultural beliefs are major constituents that can affect PTSD symptom expression (Hall-Clark et al., 2016), as well as resources to cope with the effects of trauma (Pole et al., 2005). Lastly, cross-sectional design implies that conclusions on causal relationships cannot be drawn. The mediation results should be viewed as attempts to examine the inter-correlational structure of the model rather than to explore causal relationships for which longitudinal studies are needed.

To conclude, teachers who have developed PTSD from trauma can experience difficulty in forgiving oneself or others, heightening their level of burnout. The effect of the trauma can impact their psychological well-being independently of their unforgiving attitude.

## Data Availability Statement

The raw data supporting the conclusions of this article will be made available by the authors, without undue reservation, upon request to the corresponding author.

## Ethics Statement

The studies involving human participants were reviewed and approved by the Survey and Behavioral Research Ethics Committee. The patients/participants provided their written informed consent to participate in this study.

## Author Contributions

YW, SF, and MC contributed to the design and implementation of the research, to the analysis of the results, and to the writing of the manuscript. All authors contributed to the article and approved the submitted version.

## Conflict of Interest

The authors declare that the research was conducted in the absence of any commercial or financial relationships that could be construed as a potential conflict of interest.

## Publisher’s Note

All claims expressed in this article are solely those of the authors and do not necessarily represent those of their affiliated organizations, or those of the publisher, the editors and the reviewers. Any product that may be evaluated in this article, or claim that may be made by its manufacturer, is not guaranteed or endorsed by the publisher.
